# Müller cells and retinal axons can be primary targets in experimental neuromyelitis optica spectrum disorder

**DOI:** 10.1111/cen3.12345

**Published:** 2017-01-11

**Authors:** Bleranda Zeka, Hans Lassmann, Monika Bradl

**Affiliations:** ^1^ Department for NeuroimmunologyCenter for Brain ResearchMedical University ViennaViennaAustria

**Keywords:** aquaporin 4, Müller cells, neuromyelitis optica spectrum disorder, retinal damage

## Abstract

Recent work from our laboratory, using different models of experimental neuromyelitis optica spectrum disorder (NMOSD), has led to a number of observations that might be highly relevant for NMOSD patients. For example: (i) in the presence of neuromyelitis optica immunoglobulin G, astrocyte‐destructive lesions can be initiated by CD4+ T cells when these cells recognize aquaporin 4 (AQP4), but also when they recognize other antigens of the central nervous system. The only important prerequisite is that the T cells have to be activated within the central nervous system by “their” specific antigen. Recently activated CD4+ T cells with yet unknown antigen specificity are also found in human NMOSD lesions. (ii) The normal immune repertoire might contain AQP4‐specific T cells, which are highly encephalitogenic on activation. (iii) The retina might be a primary target of AQP4‐specific T cells and neuromyelitis optica immunoglobulin G: AQP4‐specific T cells alone are sufficient to cause retinitis with low‐grade axonal pathology in the retinal nerve fiber/ganglionic cell layer. A thinning of these layers is also observed in NMOSD patients, where it is thought to be a consequence of optic neuritis. Neuromyelitis optica immunoglobulin G might target cellular processes of Müller cells and cause their loss of AQP4 reactivity, when AQP4‐specific T cells open the blood–retina barrier in the outer plexiform layer. Patchy loss of AQP4 reactivity on Müller cells of NMOSD patients has been recently described. Cumulatively, our findings in experimental NMOSD suggest that both CD4+ T cell and antibody responses directed against AQP4 might play an important role in the pathogenesis of tissue destruction seen in NMOSD.

## Introduction

Patients with neuromyelitis optica spectrum disorder (NMOSD) develop astrocyte destructive lesions, most commonly in the spinal cord and in the optic nerve. These lesions can be extensive, often become necrotic and form the pathological substrate for the severely disabling phenotype of this disease.^1^ Early NMOSD lesions are characterized by variable numbers of T cells, many neutrophils, eosinophils and macrophages/activated microglial cells, and by the deposition of immunoglobulins and complement factors on aquaporin 4 (AQP4)^+^ astrocytic end‐feet at the perivascular and subpial glia limitans.^2^ These observations strongly suggest that the pathogenic antibodies of NMOSD patients, the so‐called NMO‐immunoglobulin G (IgG), gain access to the central nervous system (CNS) under inflammatory conditions. Once within the CNS, these antibodies find their autoimmune target, the water channel AQP4^3^, ^4^ on the surface of astrocytes, bind to these cells, and initiate their destruction by antibody‐dependent cellular cytotoxicity mediated by FcγrIII^+^ macrophages/granulocytes^5^, ^6^ and by complement‐dependent cytotoxicity.^7^, ^8^, ^9^

We used this pathological information to create our animal model of experimental NMOSD, which is based on experimental autoimmune encephalomyelitis, an inflammatory disease of the CNS induced by intravenous or intraperitoneal injection of CNS antigen‐specific CD4+ T cells, which open the blood–brain barrier for the entry of antibodies and complement. At the time, when first clinical symptoms of experimental autoimmune encephalomyelitis show that the blood–brain barrier has been opened in the inflammatory process, we provide NMO‐IgG in the circulation of the experimental animals. Pathological changes strongly resembling those seen in human NMO are then evident 24–48 h after the NMO‐IgG injection.^7^

Using the experimental NMOSD model, we could already identify a number of important points that have to be considered for the interpretation of human NMOSD lesions:

1. In experimental NMOSD, it is not necessary that the CNS antigen‐specific CD4+ T cells recognize AQP4 in order to initiate astrocyte‐destructive lesions in the presence of NMO‐IgG. Also, T cells specific for other CNS antigens, for example, myelin basic protein or S100β, can open the blood–brain barrier for the entry of NMO‐IgG.^6^, ^7^ This is a very important point, as in the immune repertoire of NMOSD patients, expanded populations of AQP4‐ and proteolipid protein‐specific CD4+ T cells have been described, and other expanded populations of CNS antigen‐specific T cells might just not have been searched for.^10^, ^11^, ^12^
2. In experimental NMOSD, it became very clear that the CD4+ T cells initiating inflammatory CNS lesions must be locally reactivated in order to permit the entry of sufficient amounts of NMO‐IgG and complement for the induction of astrocyte destruction.^6^ Again, this is in line with findings of early active lesions in NMOSD patients, which contain activated CD4+ T cells.^6^
3. The T cell repertoire of rats^13^, ^14^, mice^15^, ^16^, ^17^ and humans^10^, ^11^, ^12^ contains AQP4‐specific CD4+ T cells, reacting against several different epitopes of the molecule.^14^ Depending on the epitope specificity, these T cells are weakly, moderately or strongly pathogenic^14^, and have in common that they not only target the CNS, but also muscles.^13^ At the time when we first noticed muscular inflammation, just a few sporadic cases of NMOSD patients with hyperCKemia had been described.^18^ In the meanwhile, many more of these patients were followed^19^, ^20^, ^21^, ^22^, ^23^, ^24^, ^25^, and a careful biopsy study of one of them clearly showed muscular inflammation in NMOSD as well.^21^ We also observed a subclinical infiltration of immune cells around collecting ducts at the cortico‐medullary junction of the kidney, but this did not (yet?) find its correlate in human NMOSD patients.^13^
4. In experimental NMOSD, highly pathogenic AQP4‐specific CD4+ T cells could be identified. These cells are specific for an AQP4 fragment encompassing the amino acids 268–285 (AQP4_268–285_), which contains two different, overlapping epitopes for recognition by autoimmune CD4+ T cells, AQP4_271–279_ and AQP4_273–281_ 14 When AQP4_268–285_‐specific T cells enter the CNS, they are locally reactivated, immigrate deeply into the CNS parenchyma, and open the blood–brain barrier for the entry of antibodies and complement.^14^ In NMO‐IgG seropositive hosts, astrocytes are then destroyed by complement‐dependent and/or antibody‐dependent cellular cytotoxicity (Fig. 1). This clearly shows that AQP4‐specific T cells are not only required for the formation of the pathogenic immunoglobulin isotype G1 (IgG_1_) of NMO‐IgG, but can also actively participate in the formation of inflammatory lesions in the CNS.^14^, ^17^ Unfortunately, to date, we do not have information about the antigen specificity of T cells found in the astrocyte‐destructive lesions of NMOSD patients. However, the observation of highly pathogenic AQP4‐specific CD4+ T cells in the immune repertoire of mice^17^ and rats^14^, and the observation of expanded AQP4‐specific T cell populations in the blood of NMOSD patients^10^, ^11^, ^12^ suggests that NMOSD patients also might have highly pathogenic AQP4‐specific CD4+ T cells in their immune repertoire.

**Figure 1:**
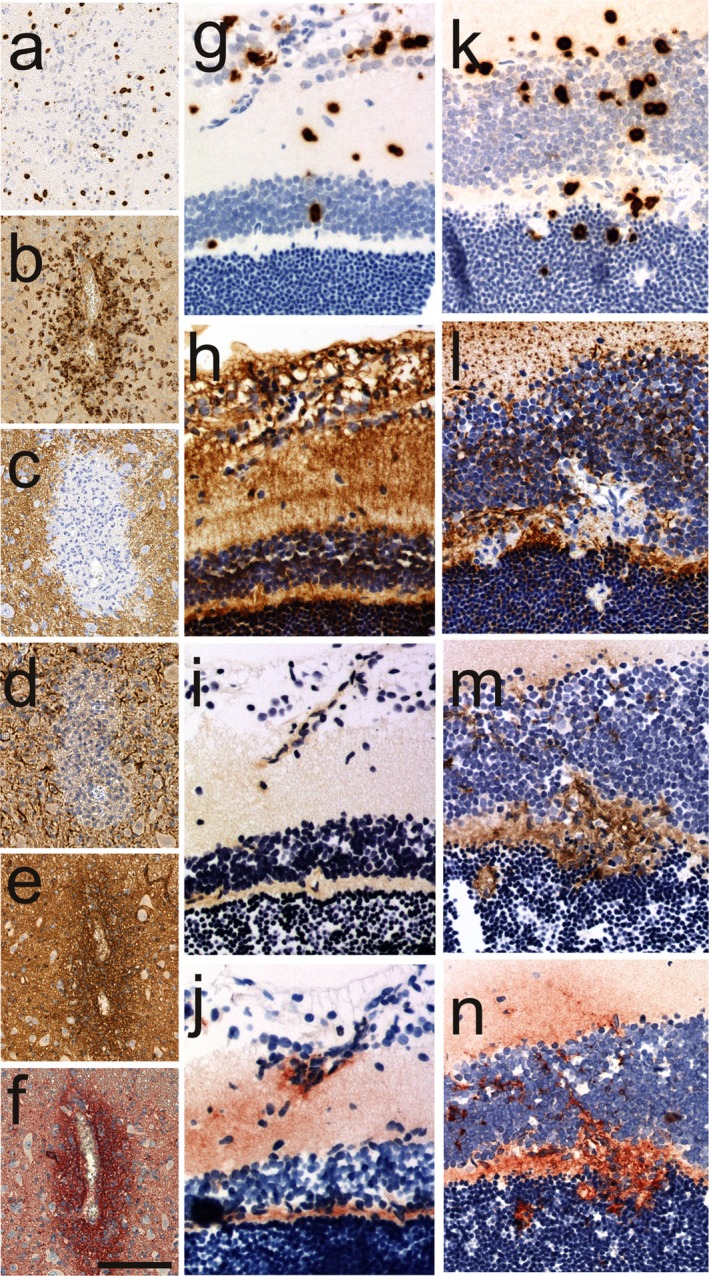
Histological analysis of inflammatory lesions in experimental neuromyelitis optica spectrum disorder induced by transfer of aquaporin 4 (AQP4) fragment encompassing the amino acids 268–285‐specific T cells and neuromyelitis optica immunoglobulin G (IgG). (a–f) Consecutive sections of a spinal cord lesion stained with (a) anti‐CD3, (b) ED1, (c) anti‐AQP4, (d) anti‐GFAP, (e) anti‐human IgG, and (f) anti‐complement C9. Note the profound loss of AQP4 reactivity, and the perivascular deposition of human IgG and complement. (g–j) Consecutive sections of a retinal T cell infiltrate coming from the retinal nerve fiber/ganglionic cell layer, stained with (g) anti‐CD3, (h) anti‐AQP4, (i) anti‐human IgG and (j) anti‐complement C9. Note that AQP4 reactivity is present, and there is only very little leakage of human IgG and complement at this site. (k–n) Consecutive sections of a retinal T cell infiltrate in the outer plexiform/inner nuclear layer, stained with (k) anti‐CD3, (l) anti‐AQP4, (m) anti‐human IgG and (n) anti‐complement C9. Note that AQP4 reactivity is lost in the outer plexiform layer, and there is profound leakage of human IgG and complement at this site. With exception of staining with anti‐complement C9 (reaction product shown in red), all other stainings show brown reaction products. All sections were counterstained to show nuclei in blue.
5. Although in experimental NMOSD induced by AQP4_268–285_‐specific T cells and NMO‐IgG, astrocytes in the spinal cord, brain and optic nerve can become targets of pathogenic antibody responses under inflammatory conditions, the largest and most frequently observed astrocyte‐destructive lesions were found in the spinal cord (Fig. 1), whereas lesions in the optic nerve are exceedingly rare and very small. This can in part be explained by the preferential infiltration of spinal cord by rat CNS antigen specific CD4+ T cells, and in part by the genetic background of our experimental animals: we used Lewis rats, which also showed much fewer optic nerve lesions than BN or DA rats when antibody and T cell responses were directed against myelin oligodendrocyte glycoprotein.^26^ A genetic contribution to lesion location has also been seen in the study of human NMOSD patients.^27^
6. Animals with experimental NMOSD induced by AQP4_268‐285_‐specific T cells and NMO‐IgG develop retinitis.^28^
  Most of the T cells extravasate from the inner blood–retinal barrier into the nerve fiber/ganglion cell/inner plexiform layers (Fig. 1), whereas a lower number of T cells enter the retina in the outer nuclear/outer plexiform/inner nuclear layers (Fig. 1). T cells in the retinal nerve fiber/ganglionic cell layer are associated with the formation of amyloid precursor protein (APP)^+^ axonal spheroids/end bulbs indicative of axonal pathology.^28^ This pathology is independent of the presence of NMO‐IgG, and is mediated by activated microglia/macrophages expressing inducible nitric oxide synthase. In NMOSD patients, a thinning of the retinal nerve fiber/ganglionic cell layers is often observed when the patients have a history of optic neuritis.^29^ Therefore, it is generally assumed that this thinning in NMOSD patients is a secondary consequence of optic neuritis. However, we saw much more axonal spheroids in the retina and papilla than in the extra‐ocular parts of the optic nerve of our experimental NMOSD models, although our animals clearly had optic neuritis as well. This suggests that primary retinal damage can occur in addition to optic neuritis.^28^ In contrast to NMOSD patients, the optic neuritis in our experimental NMOSD model was not associated with astrocyte destruction in the optic nerves. Nevertheless, our observation might also be very important for NMOSD patients, as retinal nerve fiber/ganglionic cells might be targeted twice in the disease process: by an inflammatory milieu created by AQP4‐specific T cells in the retina, causing mostly transient axonal dysfunction; and by inflammation of the optic nerve, causing secondary neurodegeneration.
7. Animals with experimental NMOSD induced by AQP4_268–285_‐specific T cells and NMO‐IgG show loss of AQP4 reactivity on Müller cells.^28^

We have shown that T cells enter the retina from two different sites: from vessels in the nerve fiber/ganglion cell/inner plexiform layers (Fig. 1), and from vessels spanning through the outer nuclear/outer plexiform/inner nuclear layers (Fig. 1). At both of these entry sites, the blood–retinal barriers are opened for the entry of NMO‐IgG and complement. Yet, we observed two radically different outcomes for pathology: AQP4^+^ retinal astrocytes, which are almost exclusively located in the nerve fiber/ganglion cell layer, and the AQP4^+^ funnel‐shaped end‐feet of Müller cells also located within this layer remained intact, and there was no indication of AQP4 loss at this site. However, the branch processes of Müller cells in the outer plexiform layer lost their AQP4 reactivity.

This effect was seen with NMO‐IgG from different patients, but not in the absence of NMO‐IgG, and it was not associated with deposition of C9 or human IgG on the surface of Müller cell bodies or stem processes. Furthermore, the Müller cells had intact nuclei, and were seemingly undamaged. Based on these observations, we concluded that the loss of Müller cell AQP4 reactivity might occur independently of complement‐dependent or antibody‐dependent cellular cytotoxicity, and speculate that the loss of AQP4 reactivity by Müller cells is caused by cross‐linking and enhanced internalization of AQP4.^28^ It is interesting to note that loss of AQP4 reactivity has also been recently described in three retinae from NMOSD autopsy cases.^30^ Also in that study, the authors described a scattered loss of AQP4 reactivity, and concluded that these pathological process might not involve complement‐dependent or antibody‐dependent cellular cytotoxicity.^30^

We do not know yet the triggers for the site‐specific differences in retinal AQP4 loss. We found more IgG and more complement in inflammatory lesions located within the outer nuclear/outer plexiform/inner nuclear layers. On the one hand, this could just simply be an indication of slightly different ages of the inflammatory lesions at the different sites.^28^ On the other hand, it could result from differences in the cytokine milieu of lesions in the outer nuclear/outer plexiform/inner nuclear layers and lesions in the retina nerve fiber/ganglionic cell layers. This could translate to local differences in the amount of interleukin‐1 beta, a cytokine facilitating barrier breakdown and lesion growth^31^ or of type I interferons, which might limit lesion formation.^32^ Different mechanisms of tissue injury might operate in parallel, as already shown in NMOSD patients.^33^

Future studies have to show how long the effects on Müller cells last, and what consequences the AQP4 loss by Müller cells has.

## Conflict of interest

None declared.
